# Development of a risk prediction model for bloodstream infection in patients with fever of unknown origin

**DOI:** 10.1186/s12967-022-03796-8

**Published:** 2022-12-08

**Authors:** Teng Xu, Shi Wu, Jingwen Li, Li Wang, Haihui Huang

**Affiliations:** 1 Institute of Antibiotics, Huashan Hospital, Fudan University, Shanghai, 200040 China; 2 Key Laboratory of Clinical Pharmacology of Antibiotics, National Health and Family Planning Commission, Shanghai, 200040 China

**Keywords:** Bloodstream infection, Fever of unknown origin, Serum inflammatory marker, Scoring system, Diagnostic performance

## Abstract

**Background:**

Bloodstream infection (BSI) is a significant cause of mortality among patients with fever of unknown origin (FUO). Inappropriate empiric antimicrobial therapy increases difficulty in BSI diagnosis and treatment. Knowing the risk of BSI at early stage may help improve clinical outcomes and reduce antibiotic overuse.

**Methods:**

We constructed a multivariate prediction model based on clinical features and serum inflammatory markers using a cohort of FUO patients over a 5-year period by Least Absolute Shrinkage and Selection Operator (LASSO) and logistic regression.

**Results:**

Among 712 FUO patients, BSI was confirmed in 55 patients. Five independent predictors available within 24 h after admission for BSI were identified: presence of diabetes mellitus, chills, C-reactive protein level of 50–100 mg/L, procalcitonin > 0.3 ng/mL, neutrophil percentage > 75%. A predictive score incorporating these 5 variables has adequate concordance with an area under the curve of 0.85. The model showed low positive predictive value (22.6%), but excellent negative predictive value (97.4%) for predicting the risk of BSI. The risk of BSI reduced to 2.0% in FUO patients if score < 1.5.

**Conclusions:**

A simple tool based on 5 variables is useful for timely ruling out the individuals at low risk of BSI in FUO population.

**Supplementary Information:**

The online version contains supplementary material available at 10.1186/s12967-022-03796-8.

## Introduction

Bloodstream infection (BSI) remains a common and important cause of death in infected patients. Population-based studies estimated that there were 575,000–677,000 episodes of BSI and 79,000–94,000 deaths per year in North America, more than 1,200,000 episodes of BSI and 157,000 deaths per year in Europe [1, 2]. A good progress has been achieved in the diagnosis and treatment of BSIs, but the mortality associated with some pathogens such as methicillin-resistant *Staphylococcus aureus*, vancomycin-resistant enterococci, carbapenem-resistant *Enterobacteriaceae* is still high [3].

The causes of fever of unknown origin (FUO) have shifted during the past century, but infections remain the leading causes of FUO [4]. BSI may also be a cause underlying FUO. In West China Hospital, 7.13% of the FUO in the patients older than 14 years of age were finally confirmed as BSI [5]. It was also reported that in West Australia, bacteremia was prevalent in 15% of the FUO patients, and 16% of the deaths of FUO patients were attributed to BSI [6]. The diagnosis of BSI poses an especially great challenge in case of FUO. A patient suffering from protracted FUO is usually treated with broad-spectrum antimicrobial agents empirically, which may suppress the growth of fastidious pathogens and confer a predisposition to antibiotic resistance. Therefore, inappropriate empiric antimicrobial therapy may further impede the early diagnosis of BSI in case of FUO, and thus delay effective therapy.

It may be helpful to improve patient outcomes and reduce antibiotic overuse if the clinicians can know the risk of BSI at early stage in FUO patients. Serum inflammatory markers such as C-reactive protein (CRP) and procalcitonin (PCT) are generally used to diagnose BSI and assess the treatment effectiveness in clinical practice [7]. These markers perform well with high sensitivity but poor specificity. DNA-based diagnostics, such as microbial next-generation sequencing (mNGS), which can rapidly detect multiple pathogens, are unfortunately expected to be available only in high-income settings and its real-world performance for BSI diagnosis has been questioned [8].

To our knowledge, few studies have assessed the risk of BSI in FUO cases and no models have been specifically developed for predicting the risk of BSI in FUO patients. In the present study, we aimed to develop a simple scoring system for predicting the risk of BSI in FUO patients, which can be calculated from readily available routine clinical variables within 24 h after admission.

## Methods

### Study design

The FUO patients were consecutively enrolled for analysis at Huashan Hospital, Fudan University, a 2000-bed tertiary care teaching hospital in Shanghai, China. The cohort consisted of retrospectively enrolled patients between January 2014 and June 2017 and those prospectively enrolled between December 2017 and May 2019].

Ethical approval was granted by the Institutional Review Board of Huashan Hospital, Fudan University for prospectively collecting the patient data. All the prospectively enrolled patients signed the informed consent form before data collection. This cohort study was registered at Chinese trial register (www.chictr.org.cn, ChiCTR1800020037) [9]. The principles for transparent reporting of a multivariable prediction model for individual prognosis or diagnosis (TRIPOD) were observed in this study [10] (Additional file 2).

### Study population

All of the enrolled patients were adults (at least 18 years of age) satisfying the criteria for classic FUO, i.e., body temperature above 38.3 °C on three or more occasions and at least 3-week duration of illness, in which no diagnosis was made after 1 week of hospital admission [11, 12]. The patients were excluded in case of: (1) pregnant; (2) readmission within 3 months after discharge; (3) discharge from hospital before identifying FUO etiology; or (4) missing data of CRP, PCT levels or other relevant test results within 24 h after admission.

BSI was defined by positive blood cultures in a patient with systemic signs of infection, excluding bacteria such as *Corynebacterium* species which were often associated with blood culture contamination. However, multiple positive cultures for common commensals (e.g., coagulase-negative *Staphylococcus*) were considered true BSI events if the second culture occurred on the same or subsequent day [13].

### Data collection

The following clinical and laboratory data were collected, including patient demographics, clinical features, comorbidities, laboratory test results, patient outcomes, and discharge diagnoses. Invasive procedure included endoscopy, puncture, tissue biopsy, hemodialysis and acupuncture. Catheter use including urinary catheter, peripherally inserted central catheter, drainage tube, nasobiliary duct and gastric tube. Implantations included double J tubes, pacemakers, artificial joints, heart valves, artificial ossicles, coronary stents, inferior vena cava filters and fracture internal fixation devices. Corticosteroid use referred to receiving an equivalent dose of ≥ 15 mg prednisone per day; chemotherapy referred to receiving cytotoxic anti-tumor drugs to cure or alleviate cancer.

### Laboratory tests

The results of blood culture within 48 h of admission were also recorded and carefully evaluated. CRP and PCT (Upper Bio-Tech Pharma, Shanghai, China) were measured for all the prospectively enrolled FUO patients within 24 h after admission per the manufacturer’s instruction. The CRP and PCT data within 24 h after admission were retrieved from the electronic case record for the retrospectively enrolled FUO patients.

### Statistical analysis

The continuous random variables were firstly evaluated by Kolmogorov–Smirnov test to verify the normality of their distribution, then compared between groups by Student *t*-test or Mann–Whitney U-test. The categorical variables were compared between groups by Pearson’s Chi-square test or Fisher’s exact test. The difference was considered statistically significant when the two-sided p value was < 0.05.

The missing data for a variable were subjected to imputation when no more than 20%, otherwise, the variable was removed from the analysis. Predictive mean matching (PMM), Logistic regression, and Bayesian polytomous regression methods were used to impute the missing values for continuous, dichotomous, and categorical variables, respectively. The continuous random variables were evaluated by restricted cubic splines with four knots at the 5th, 35th, 65th, and 95th percentiles of the distributions for their possible non-monotonic relationship to the dependent variable (BSI). The variables monotonously related to BSI were converted to binary variables according to the best cutoff value. Those non-monotonously related variables were converted to multi-category variables according to the spline curve graph. Considering the relatively small number of observed events in this study, least absolute shrinkage and selection operator (LASSO) regression augmented with tenfold cross validation was adopted to minimize the potential collinearity of variables measured from the same patient and over-fitting of variables, which used the λ operator to penalize the absolute value of regression coefficients. Specifically, the larger the value of λ operator would shrink the smaller absolute value of regression coefficient to zero, leaving only the strongest predictors. For model concision, a value of λ with five predictors left for variable screening were selected for this study. The variables identified by LASSO regression analysis were subsequently entered into logistic regression. The statistically significant variables were used to construct the final predictive model.

The receiver operating characteristic (ROC) curve and the corresponding area under the curve (AUC) were used to evaluate model discrimination. Hosmer–Lemeshow test was used to evaluate the model calibration. After internal validation via 1000 repetitions of Bootstrap resampling, the resulted regression model was further simplified to a scoring system according to the method previously described [14]. Calibration plot was used to assess the goodness of fit. A schematic diagram comparing pre- and post-test probability of BSI was presented to assess the clinical utility of the scoring system [15]. All statistical analyses were performed using R statistical software (version 4.0.2, R Foundation) and SPSS software (version 25.0, IBM).

## Results

Overall, 527 retrospective FUO patients and 185 prospective FUO patients were enrolled. Fifty-five cases of BSI were identified, including 48 in the retrospective cohort, and 7 in the prospective cohort. The cohort of 712 FUO patients and 55 BSI patients were used to develop the prediction model.

The patients with BSI had higher body temperature than the patients without BSI, and associated more frequently with chills, diabetes mellitus, liver cirrhosis, solid tumor, catheter use, and implant device (Table 1). Single pathogen was finally isolated from each of the 55 patients with BSI by blood culture, including *Escherichia coli* (36.3%), *Streptococcus* spp. (10.9%), and *Klebsiella pneumoniae* (10.9%) (Additional file 1: Table S1).

**Table 1:** Demographics and clinical characteristics of the patients for developing the prediction model

Characteristic	Total	Bloodstream infection		*P* value
		Yes	No	
Number of patients	712	55	657	NA
Demographics				
Age, median (IQR), yrs	52 (33–65)	56 (38–66)	52 (33–65)	0.201
Male sex	364 (51.1)	29 (52.7)	335 (51.0)	0.804
Final diagnosis				
Infectious disease	366 (51.4)	55 (100)	311 (47.3)	NA
NIID	203 (28.5)	NA	203 (30.9)	NA
Malignancies^a^	52 (7.3)	NA	52 (7.9)	NA
Other and unknown	91 (12.8)	NA	91 (13.9)	NA
Comorbidity				
Diabetes mellitus	72 (10.1)	17 (30.9)	55 (8.4)	0.000
Liver cirrhosis	12 (1.7)	4 (7.3)	8 (1.2)	0.001
Chronic kidney disease	14 (2.0)	3 (5.5)	11 (1.7)	0.062
Solid tumor	24 (3.4)	7 (12.7)	17 (2.6)	0.001
Hematological malignancy	7 (1.0)	1 (1.8)	6 (0.9)	0.432
Other underlying condition				
Corticosteroid use^b^	17 (2.4)	3 (5.5)	14 (2.1)	0.137
Chemotherapy^b^	9 (1.3)	2 (3.6)	7 (1.1)	0.149
Invasive procedure^b^	63 (8.8)	9 (16.4)	54 (8.2)	0.049
Catheter use	15 (2.1)	4 (7.3)	11 (1.7)	0.023
Implant device	12 (1.7)	4 (7.3)	8 (1.2)	0.010
Clinical feature				
Highest temperature, median (IQR), ℃	39.2 (38.8–39.9)	39.5 (39.0–40.0)	39.2 (38.8–39.8)	0.004
Chills	208 (29.2)	32 (58.2)	176 (26.8)	0.000
Outcome				
In-hospital mortality	0 (0)	0 (0)	0 (0)	NA
LOS, median (IQR), d	11 (7–17)	14 (10–21)	11 (7–17)	0.004

Data are presented as *n* (%) unless otherwise indicated

*IQR* interquartile range, *LOS* length of stay, *NA* not applicable/available; *NIID* non-infectious inflammatory disease

^a^Tumor-related fever should meet all the following conditions: there is no evidence of infection based on physical examination, laboratory (including sterile body fluid smear and culture) and imaging examination. Antibiotic treatment did not reduce the highest body temperature. The highest body temperature was really reduced after treatment with non-steroidal anti-inflammatory drugs. Otherwise, the tumor is regarded as a comorbidity rather than a cause of fever

^b^Within 30 days before admission

### Performance of serum inflammatory markers in identifying BSI

**Fig. 1:**
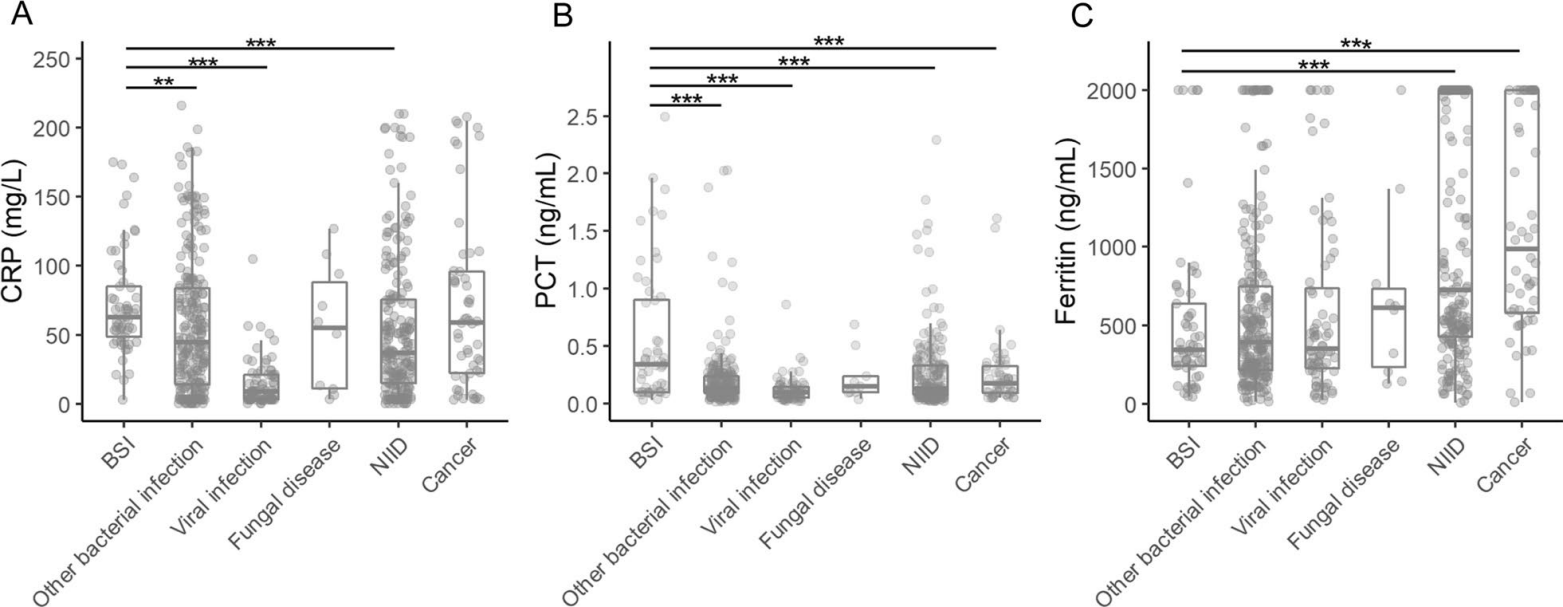
Serum levels of CRP (**A**), PCT (**B**) and serum ferritin (**C**) in patients with fever of unknown origin within 24 h after admission. *BSI* bloodstream infection, *CRP* C-reactive protein, *NIID* non-infectious inflammatory disease, *PCT* procalcitonin. *p < 0.05, **p < 0.01, ***p < 0.001

**Fig. 2:**
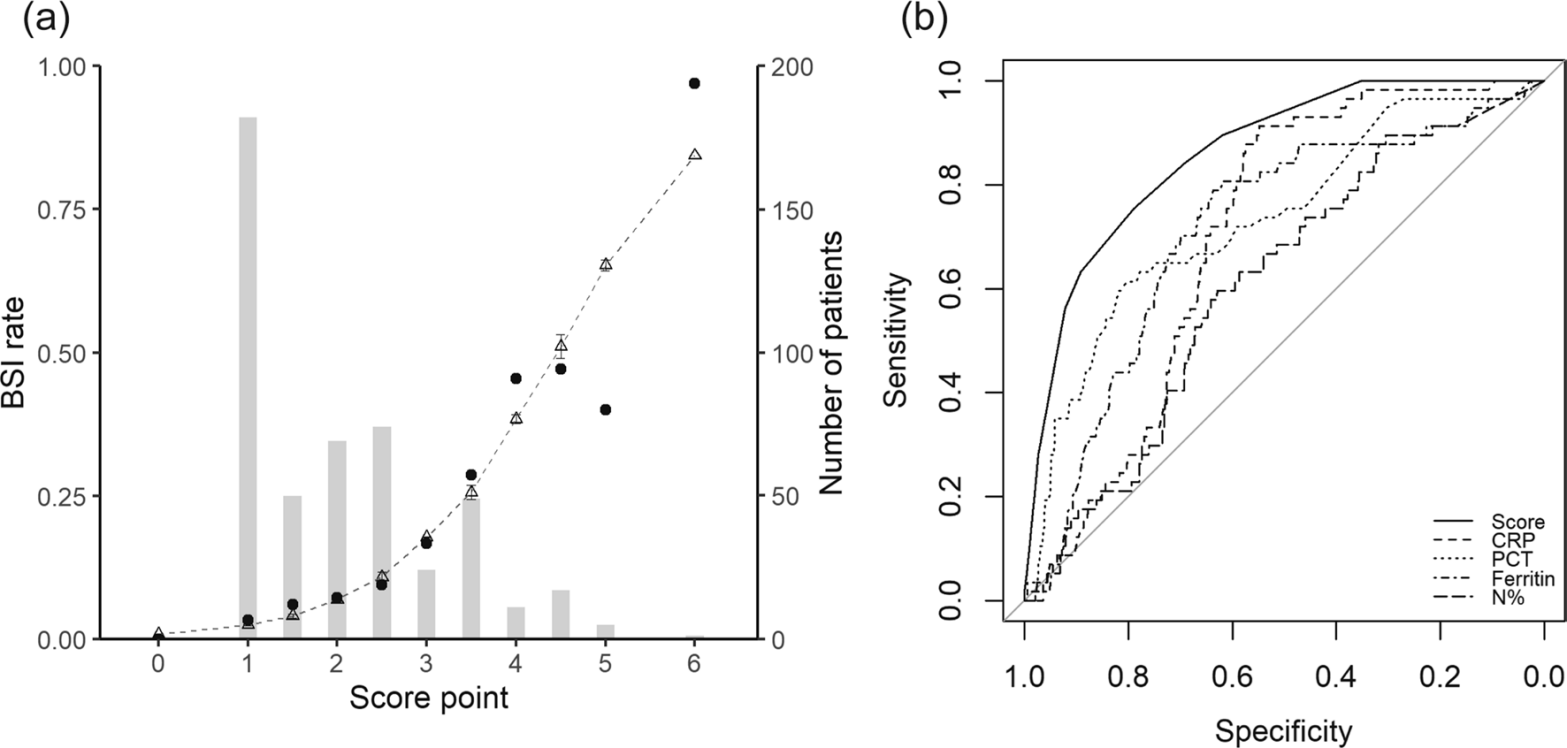
Calibration and discrimination of the risk score for BSI in FUO patients. The predicted (mean ± SD) rates of bloodstream infection (triangles and dotted line) at each risk score vs. observed rates of BSI (solid circles) are presented in **a**. Grey bars show the number of patients (right Y-axis) analyzed per score. Discrimination of the BSI status by risk score using area under the receiver operating curve analysis are presented in **b**. *BSI* bloodstream infection, *CRP* C-reactive protein, *FUO* fever of unknown origin, *PCT* procalcitonin, *SD* standard deviation

The levels of CRP (Fig. 1A), PCT (Fig. 1B) and serum ferritin (Fig. 1C) within 24 h after admision are dpicted. BSI was associated with significantly higher CRP (62.80 versus 34.30 mg/L, p < 0.001) and PCT (0.38 versus 0.12 ng/mL, p < 0.001) levels, but significantly lower ferritin level (341.50 versus 535.40 mg/L, p < 0.05) compared with the FUO patients without BSI. The AUC of CRP, PCT, and ferritin for diagnosing BSI was 0.70 (95% CI 0.65–0.75), 0.73 (95% CI 0.66–0.80), and 0.62 (95% CI 0.55–0.69), respectively (Fig. 2B).

### Development and utility of the prediction model

The continuous variables age, CRP, and erythrocyte sedimentation rate (ESR) showed non-monotonic U shape association with BSI. These variables were converted to 3-category variables for analysis (Additional file 1: Fig. S1). Only 5 strong predictors remained in the prediction model when the λ of LASSO regression was 0.0415, i.e., presence of diabetes mellitus and chills, neutrophil percentage > 75%, initial CRP level of 50–100 mg/L, and procalcitonin > 0.3 ng/mL within 24 h after admission (Additional file 1: Fig. S2). Multivariate analysis demonstrated that these variables were also independent predictors of BSI (Table 2).

**Table 2:** Multivariate logistic regression model and scoring system for predicting the risk of BSI in adult patients with fever of unknown origin

Variable	β-coefficient	*OR* (95% CI)	*P* value	Score (point)
Diabetes mellitus	1.70	5.46 (2.62–11.39)	0.000	1.5
Chills	1.13	3.11 (1.66–5.80)	0.000	1
C-reactive protein, mg/L				
≤ 50	Reference	Reference	Reference	0
50–100	1.04	2.83 (1.40–5.73)	0.004	1
> 100	0.01	1.00 (0.41–2.47)	0.998	0
Procalcitonin > 0.3 ng/mL	1.52	4.58 (2.41–8.70)	0.000	1.5
Neutrophil percentage > 75%	1.06	2.87 (1.49–5.54)	0.002	1

A logistic regression model based on these five predictors showed good discrimination (C-statistic, 0.85) and calibration (Hosmer–Lemeshow Chi-square statistic, 7.02; p > 0.05). Bootstrap simulation suggested no significant overoptimism (optimism-corrected C-statistic, 0.83; calibration slope, 0.91).

**Fig. 3:**
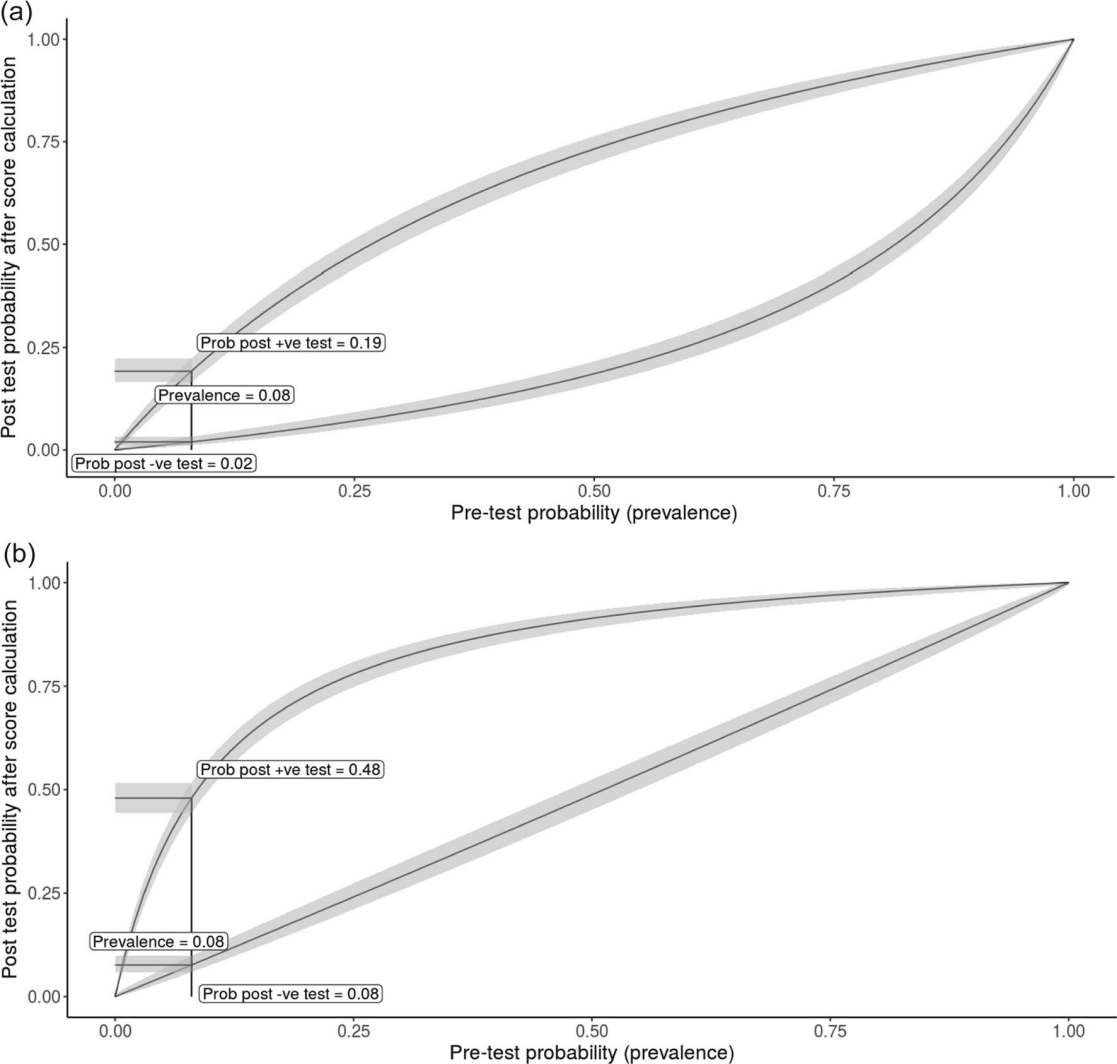
Pre- and post-test probability for the scoring system in diagnosing bacterial infections in patients with fever of unknown origin. Post-test probability was calculated as pre-test odds × likelihood ratio. Cut-off value = 1.5 points in **A**. Cut-off value = 4.5 points in **B**. −ve: negative; +ve: positive; Prob: probability

Each of these five independent variables was scored according to the corresponding β-coefficient of the variable. The sum of the score was calculated as the risk score of BSI for an individual patient, which ranged from 0 to 6 points (Table 2). The risk score (median, interquartile range [IQR]) was significantly higher among the FUO patients with BSI (3.5, IQR [2.0–4.0]) compared with those without BSI (1, IQR [0.0–2.0]; p < 0.001). The incidence rate of BSI predicted by the risk score was well calibrated with the observed rate (Fig. 2A). This scoring tool could identify BSI appropriately, evidenced by an AUC of 0.85 (95% CI 0.80–0.90) (Fig. 2B). The optimal cut-off value of this scoring system was 2.5 points. Its performance in predicting the risk of BSI among the FUO cohort showed good sensitivity (74.5%) and specificity (78.7%), low positive predictive value (22.6%), but excellent negative predictive value (97.4%). The positive and negative likelihood ratios (LRs) were 3.5 and 0.32, respectively. The pre-test probability (prevalence) of BSI in this FUO cohort was 8.0%. When the risk score is < 1.5, the risk of BSI decreases to 2%, when the risk score is ≥ 4.5, the risk of BSI increases to 48% (Fig. 3).

## Discussion

We developed a novel practical diagnostic instrument to predict the risk of BSI in FUO patients following the TRIPOD guidance. To our knowledge, no such prediction tool has been reported specifically for the FUO patients. The scoring system consisted of five variables routinely assessed for FUO patients in clinical practice: diabetes mellitus, chills, CRP, PCT, and neutrophil percentage. These variables are readily tested or available to clinicians for calculating the risk score of BSI. The newly developed scoring tool was internally validated and showed good discrimination and calibration abilities.

Previous studies have shown that the commonly used inflammatory markers such as PCT and CRP alone are not accurate enough in identifying BSI [16–18]. PCT generally showed higher predictive value for BSI than CRP, but its AUC varied (0.70–0.85) when used in different wards and populations [19–21]. In our FUO cohort, PCT level > 0.3 ng/mL within 24 h after admission alone provided only a moderate AUC (0.73, 95% CI 0.66–0.80) in identifying BSI, with high specificity to discriminate BSI (80.1%) but low sensitivity (60.0%). CRP alone for diagnosing BSI is far from satisfactory [22]. Serum CRP > 150 mg/L was not an independent risk factor for BSI in febrile adults in emergency department [14]. A retrospective study of FUO population also revealed a higher median level of CRP in connective tissue diseases than in infectious diseases [23]. It’s worth noting that a U-shape relation between CRP and BSI were observed in our cohort (Additional file 1: Fig. S1). More than half (50.9%) of the patients with BSI had CRP levels between 50 and 100 mg/L. However, CRP > 100 mg/L was reported in more cases of non-infectious inflammatory diseases (38 cases) and other bacterial infections (41 cases) than in BSIs (12 cases).

Some authors have used the prediction model combining risk factors and clinical manifestations with inflammatory markers to identify BSI at early stage. Su et al. developed a scoring tool to predict BSI for the patients (> 15 years of age) in the emergency department [24]. Their scoring system adopted serum markers CRP (> 10 mg/dL) and PCT (> 0.5 ng/mL) in addition to the clinical predictors such as fever and sinus tachycardia. The reported AUC for this tool was 0.85 in diagnosing BSI, and 0.79 even in the febrile subset. However, the risk factors of BSI such as invasive procedures and indwelling catheter were not considered as the variables for screening in constructing their model. Recently, some researchers have also used machine learning algorithms to predict BSI. The results varied greatly with the variables included, algorithms, and target populations. Ratzinger et al developed a stochastic forest model using 29 parameters including clinical variables, routine laboratory tests, and cytokines for diagnosing bacteremia in the systemic inflammatory response syndrome (SIRS) population in standard care wards, which resulted in an AUC of 0.74 [25]. Roimi et al. developed a prediction model using the datasets of ICU patients in two hospitals. The model included 50 clinical and laboratory variables. The diagnostic performance (AUC) was 0.87 and 0.93 in internal validation [26]. However, the machine learning approach requires that an electronic database containing all the patient characteristic parameters is available for the algorithm to work, so it is not readily applicable to most of the healthcare settings. In the present study, the scoring system was designed for FUO patients specifically. It is a simple and practical instrument that can be used at bedside. The predictors are well defined, easily measured, and readily available within 24 h after patient admission. It performed well in identifying BSI in FUO patients (AUC, 0.85).

The unnecessary antimicrobial therapy increases the risk of adverse drug reactions and secondary *Clostridioides difficile* infections, and promotes the spread of antibiotic-resistant bacteria. Inappropriate antibiotic use is a common problem in FUO population. Mert et al. reviewed the records of 20 FUO patients, who finally were diagnosed as adult onset Still disease, and found that 18 of the patients received unnecessary antibiotics prescription [27]. At present, the expert consensus on the diagnosis and treatment of FUO states that FUO without agranulocytosis should not be routinely treated with empirical antimicrobial therapy [4, 28]. However, for BSI, early use of antibiotics can improve the patient outcome. Therefore, identification of an individual at high likelihood of BSI among FUO patients has the potential to help clinicians make reasonable treatment decision. This scoring tool can help clinicians assess the possibility of BSI in patients with typical FUO within 24 h of admission and decide whether anti-infective treatment needs to be initiated immediately. In our center, the pre-test probability (prevalence) of BSI is 8.0% among FUO patients. Therefore, if the score of an individual is less than 1.5 after admission, his/her risk of BSI is very low (only 2%), suggesting that doctors can consider suspending antibiotic prescription.

This study has some limitations. More than 700 FUO cases were included over 5 years, but there are still few cases of BSI. The results were from a single center and have not been externally verified. Therefore, it is required to further evaluate the utility of this scoring tool in other medical institutions. Other relevant markers such as IL-6 were also used in the diagnosis of severe infections, but they were not routinely tested for FUO patients in the study center at that time, so they were not included as possible variables in the screening. The diagnostic performance of this model is unclear for the fungal BSI in FUO cases because no fungal pathogen was isolated in this study.

In summary, a simple scoring system was constructed with a combination of five variables: diabetes mellitus, chills, CRP (50–100 mg/L), PCT (> 0.3 ng/mL), and neutrophil percentage (> 75%) in the cohort of 712 FUO patients. The tool is helpful for clinicians to identify the FUO patients at low risk of BSI, and so avoid inappropriate antibiotic use. The clinical utility of this scoring tool still needs to be confirmed by external validation with larger sample size.

## Supplementary Information

**Additional file 1: Table S1.** The organismsisolated from blood culture from 55 cases of bloodstream infection in the modeldevelopment cohort. **Figure S1.** Association betweencontinuous variables and BSI in FUO patients demonstrated by restricted cubicsplines (RCS). *BSI* bloodstream infection,*ESR* erythrocyte sedimentation rate, *FUO* fever of unknown origin, *Nper* neutrophil percentage, *temp* body temperature, *WBC* white blood cell count. **FigureS2.** Variables selectionusing the least absolute shrinkage and selection operator (LASSO) binarylogistic regression model. Panel A. LASSO coefficient profiles of the candidatevariables. Panel B. Tuning parameter (λ) selection in the LASSO model using tenfoldcross-validation via minimum criteria.

**Additional file 2.** TRIPOD checklist:prediction model development and validation.
