# Supplementary material for: Development of a risk prediction model for bloodstream infection in patients with fever of unknown origin
Source: J Transl Med. 2022 Dec 8;20:575. doi: 10.1186/s12967-022-03796-8 (PMC9733314; doi:10.1186/s12967-022-03796-8)
Supplement: Supplementary file 1 — Additional file 1: Table S1. The organismsisolated from blood culture from 55 cases of bloodstream infection in the modeldevelopment cohort. Figure S1. Association betweencontinuous variables and BSI in FUO patients demonstrated by restricted cubicsplines (RCS). BSI bloodstream infection,ESR erythrocyte sedimentation rate, FUO fever of unknown origin, Nper neutrophil percentage, temp body temperature, WBC white blood cell count. FigureS2. Variables selectionusing the least absolute shrinkage and selection operator (LASSO) binarylogistic regression model. Panel A. LASSO coefficient profiles of the candidatevariables. Panel B. Tuning parameter (λ) selection in the LASSO model using tenfoldcross-validation via minimum criteria. [file 12967_2022_3796_MOESM1_ESM.docx]

**Additional File 1.**

**Table S1**

The organisms isolated from blood culture from 55 cases of bloodstream infection in the model development cohort

| **Organisms** | **Patients, *n* (%)** |
| --- | --- |
| *Acinetobacter baumannii* | 1 (1.8) |
| *Brucella bacterium* | 2 (3.6) |
| *Enterobacter cloacae* | 2 (3.6) |
| *Enterococcus faecalis* | 5 (9.1) |
| *Enterococcus faecium* | 1 (1.8) |
| *Enterococcus gallinarum* | 1 (1.8) |
| *Escherichia coli* | 20 (36.4) |
| *Francisella tularensis* | 1 (1.8) |
| *Granulicatella adiacens* | 1 (1.8) |
| *Klebsiella pneumoniae* | 6 (10.9) |
| *Morganella morganii* | 1 (1.8) |
| *Proteus mirabilis* | 1 (1.8) |
| *Pseudomonas aeruginosa* | 1 (1.8) |
| *Salmonella* spp. | 3 (5.5) |
| *Staphylococcus aureus* | 1 (1.8) |
| *Staphylococcus hominis* | 1 (1.8) |
| *Stenotrophomonas maltophilia* | 1 (1.8) |
| *Streptococcus constellatus* | 1 (1.8) |
| *Streptococcus gallolyticus* | 1 (1.8) |
| *Streptococcus mutans* | 1 (1.8) |
| *Streptococcus oralis* | 1 (1.8) |
| *Streptococcus viridans* | 2 (3.6) |


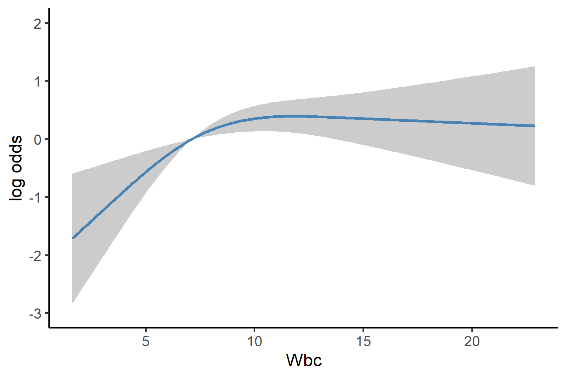

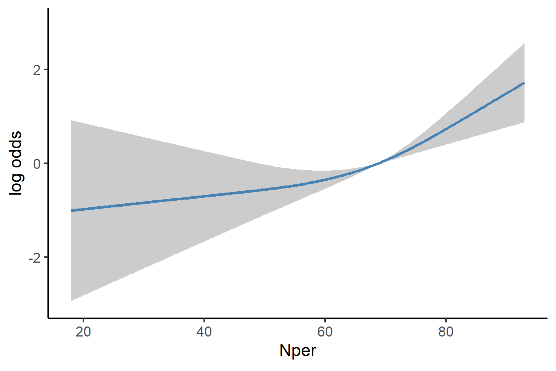


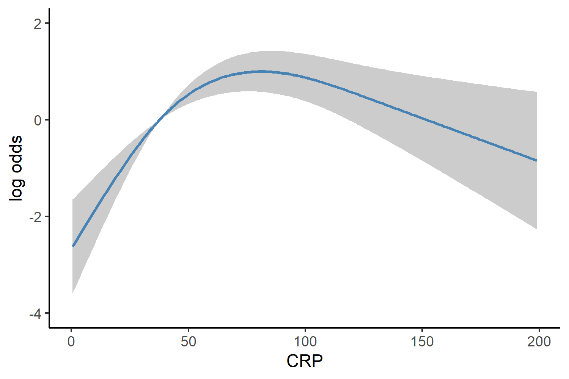

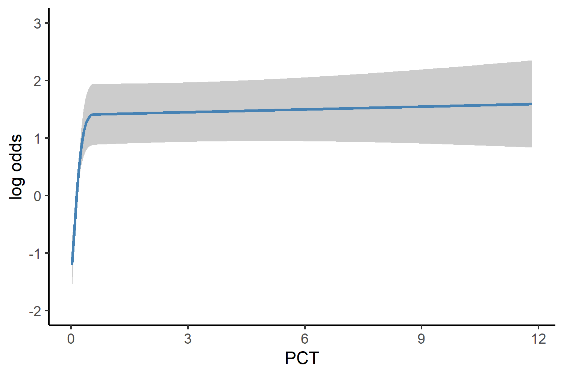


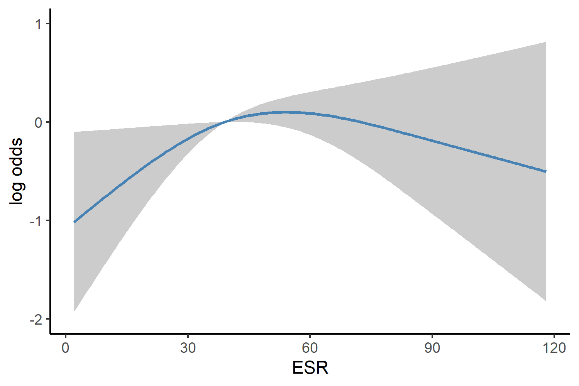

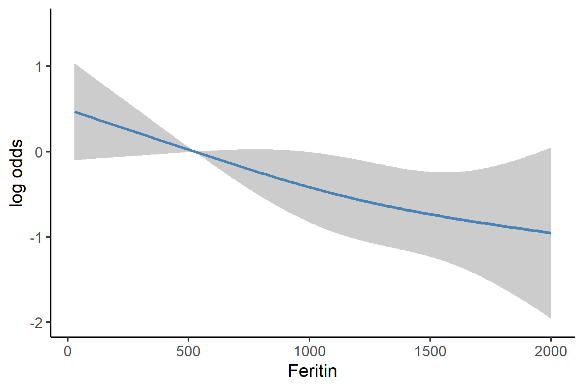


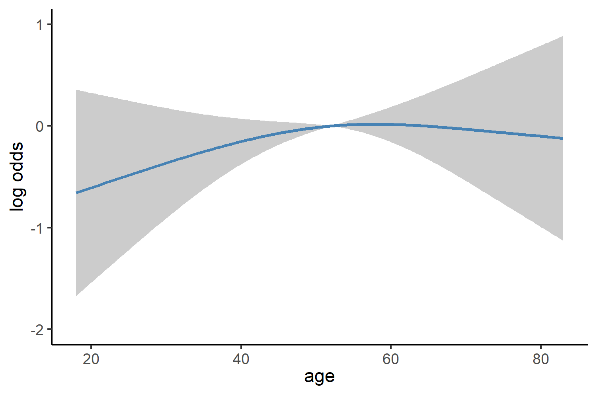

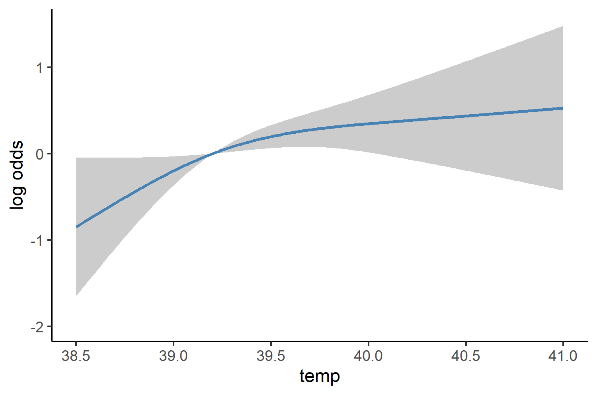


**Figure S1.** Association between continuous variables and BSI in FUO patients demonstrated by restricted cubic splines (RCS). BSI, bloodstream infetion; ESR, erythrocyte sedimentation rate; FUO, fever of unknown origin; Nper, neutrophil percentage; temp, body temperature; WBC, white blood cell count.

A.


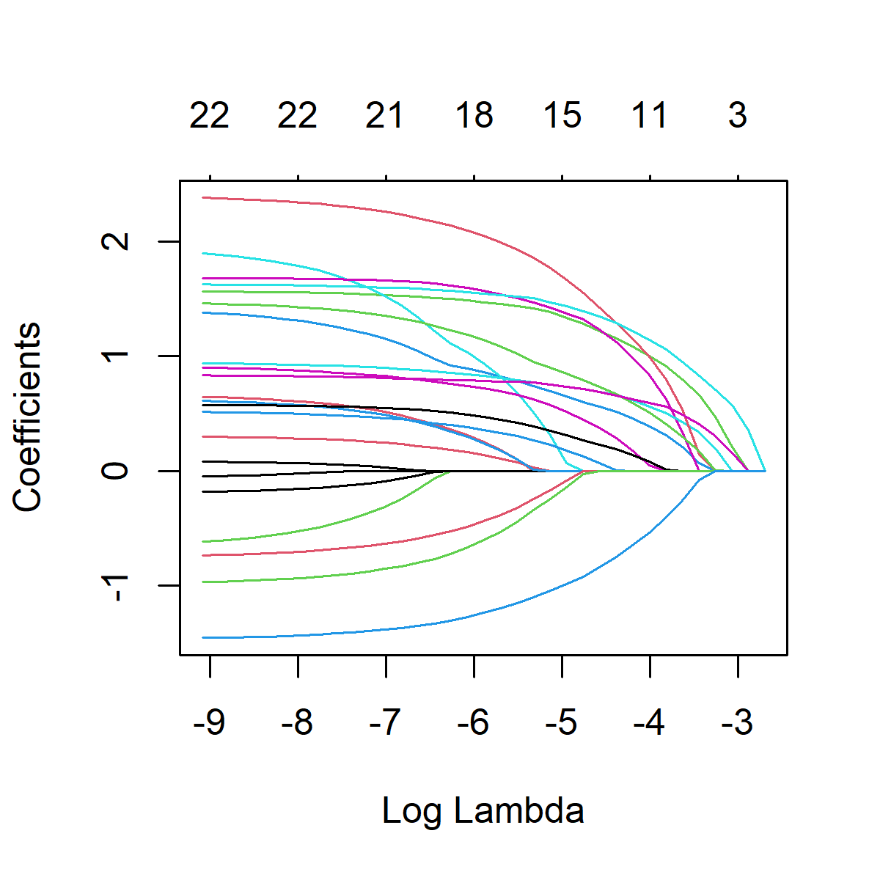


B.


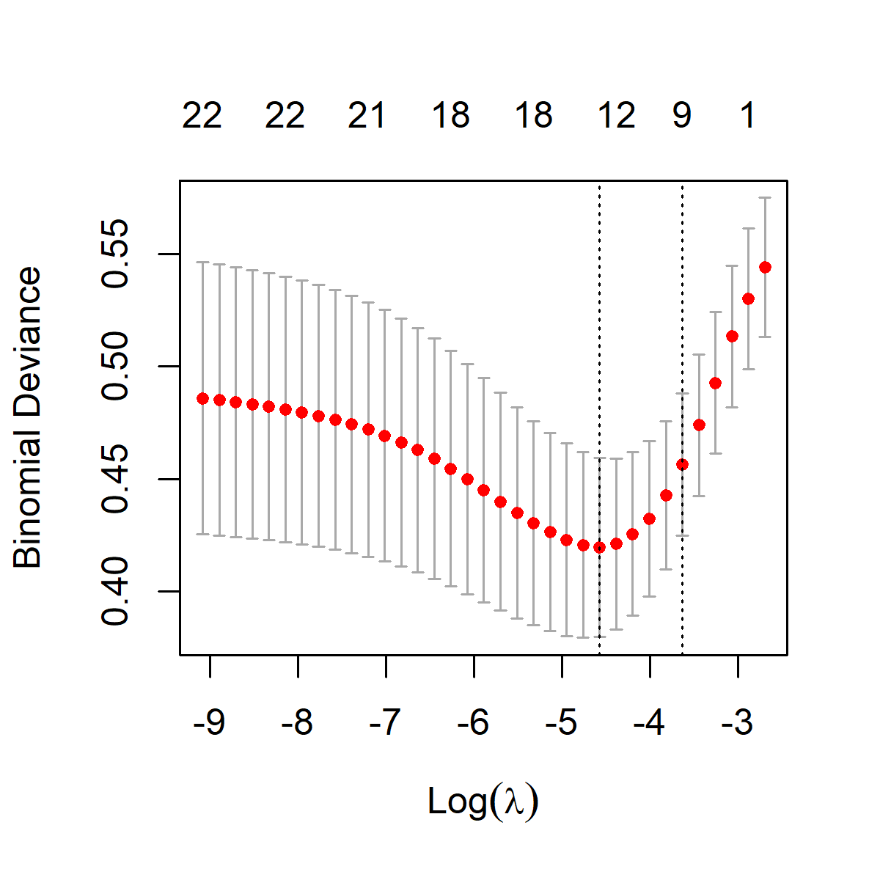


**Figure S2.** Variables selection using the least absolute shrinkage and selection operator (LASSO) binary logistic regression model. Panel A. LASSO coefficient profiles of the candidate variables. Panel B. Tuning parameter (λ) selection in the LASSO model using 10-fold cross-validation via minimum criteria.
